# 2-(10-Bromo­anthracen-9-yl)-*N*-phenyl­aniline

**DOI:** 10.1107/S2414314624004759

**Published:** 2024-05-31

**Authors:** Dhandayutham Saravanan, C. Ponraj, Themmila Khamrang, Madhukar Hemamalini, G. Jerald Maria Antony

**Affiliations:** a Department of Chemistry, National College, Thiruchirappalli, Tamil Nadu, India; b Department of Chemistry, Dhanamanjuri University, Manipur 795 001, India; c Department of Chemistry, Mother Teresa Women’s University, Kodaikanal, Tamil Nadu, India; University of Aberdeen, United Kingdom

**Keywords:** crystal structure, C—H⋯π inter­actions

## Abstract

In the title compound, C_26_H_18_BrN, the central benzene ring makes dihedral angles with its adjacent anthracene ring system and pendant benzene ring of 87.49 (13) and 62.01 (17)°, respectively. The N—H moiety is sterically blocked from forming a hydrogen bond, but weak C—H⋯π inter­actions occur in the extended structure.

## Abstract 2

The N—H group of the title compound does not form a hydrogen bond due to steric hindrance.

## Graphical abstract

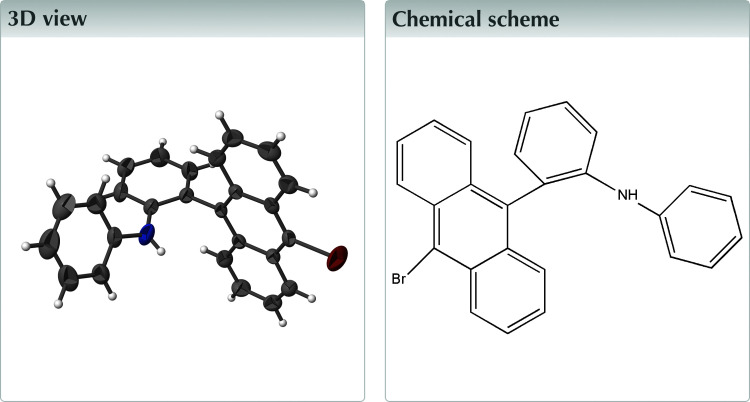

## Structure description

Anthracene derivatives are candidates for two-dimensional mol­ecular crystals, which can show inter­esting properties with applications in electronics, biomedicine, and sensors (Yan *et al.*, 2023 ▸). As part of our studies of anthracene derivatives, we now report the synthesis and crystal structure of the title compound, C_26_H_18_BrN, (**I**).

**Figure 1:**
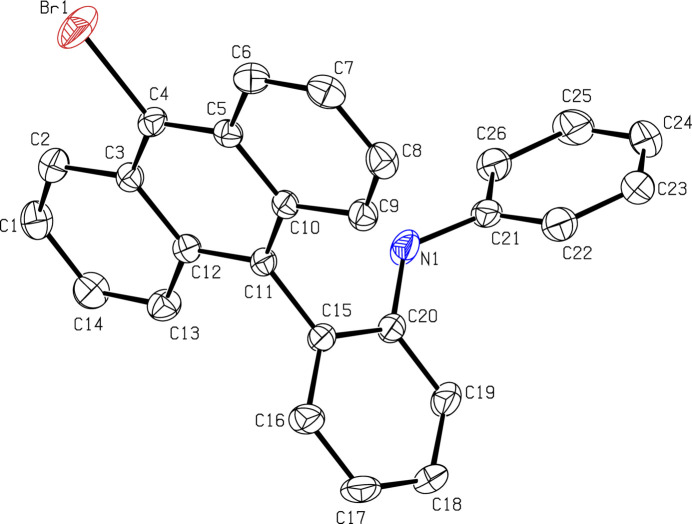
The mol­ecular structure of (**I**) showing displacement ellipsoids at the 50% probability level (H atoms are omitted for clarity).

The mol­ecular structure of (**I**) is illustrated in Fig. 1 ▸. As expected, the anthracene (C1–C14) ring system is almost planar, with a maximum deviation of 0.039 (4) Å for atom C1. The central benzene (C15–C20) ring makes dihedral angles of 87.49 (13) and 62.01 (17)° with the anthracene ring system and the terminal C21–C26 phenyl ring, respectively. The dihedral angle between the phenyl ring and anthracene ring system is 87.92 (14)°.

**Figure 2:**
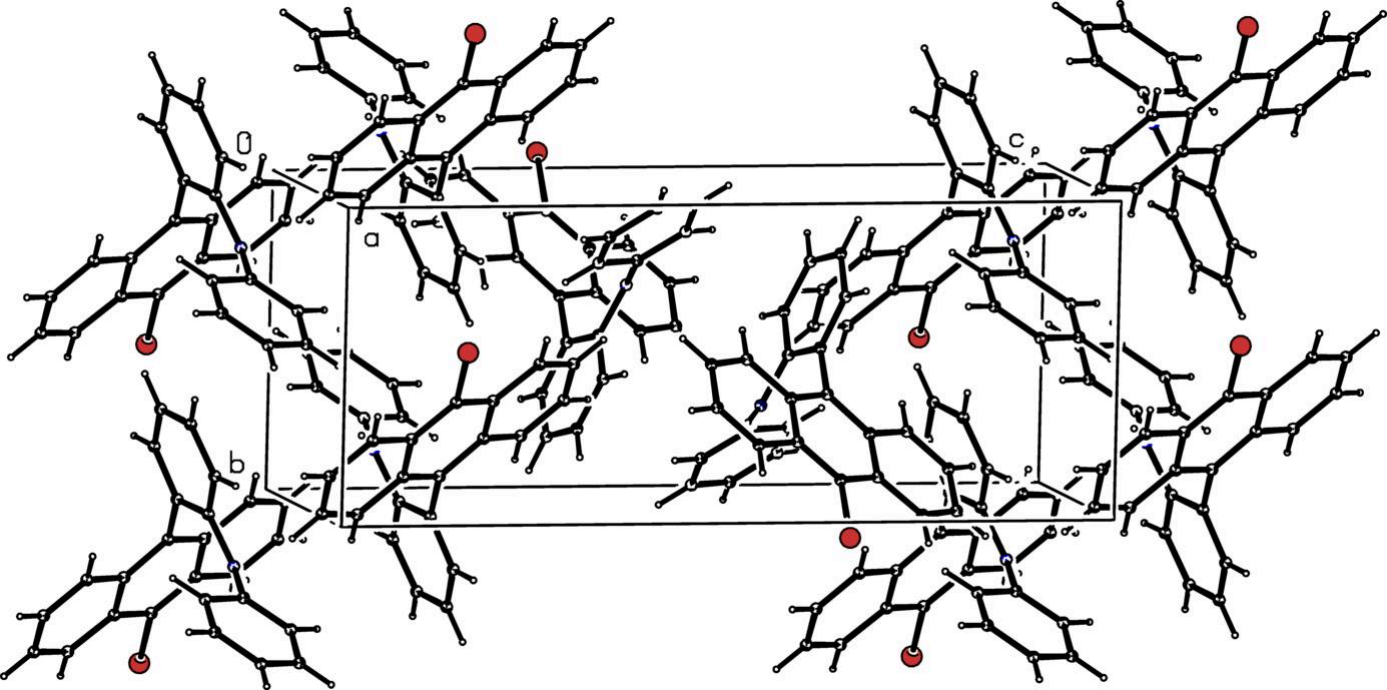
The crystal packing of (**I**) viewed approximately down [000].

In the extended structure, the N—H grouping in (**I**) is presumably blocked from forming a hydrogen bond due to steric reasons but two weak C—H⋯π inter­actions are observed (Table 1 ▸). The packing is illustrated in Fig. 2 ▸.

Related structures reported in the Cambridge Structural Database (CSD, Version 5.41, updated November 2019; Groom *et al.*, 2016 ▸) include {1-[2-(9-anthr­yl)phen­yl]-3-[2-(4-isopropyl-4,5-di­hydro-1,3-oxazol-2-yl)propan-2-yl]-1,3-di­hydro-2*H*-benzimidazole-2-thione}di­chloro­palladium(II) deutero­chloro­form solvate (CSD refcode BUVGEF; Gao *et al.* 2010 ▸), 10-bromo-2,7-di-*tert*-butyl-*N,N*-bis­(4-methyl­phen­yl) anthracen-9-amine (FEKTOG; Hoffend *et al.*, 2012 ▸) and 9-(10′-bromo-9′-anthr­yl)carbazole (PEDSUM; Boyer *et al.*, 1993 ▸).

## Synthesis and crystallization

Following the method of Justin Thomas *et al.* (2005 ▸), a mixture of di­phenyl­amine (1.69 g, 10.0 mmol), sodium *tert*-butoxide (1.15 g, 12.0 mmol) and Pd_2_(dba)_3_ (dba = di­benzyl­ideneacetone; 23 mg, 0.10 mmol) was dissolved in dry toluene (50 ml), and 9,10-di­bromo­anthracene (3.33 g, 10.0 mmol) and 1,1′-ferrocenediyl-bis­(di­phenyl­phosphine) (0.277 g, 0.5 mmol) were added sequentially. The mixture was heated to reflux, stirred for 24 h and then cooled and 5 ml of water were added. The solution was extracted with di­chloro­methane/water. The organic layer was dried over anhydrous sodium sulfate, filtered, and dried. The residue was chromatographed through silica gel using a mixture of di­chloro­methane and hexane as the eluent to give the pure product as yellow crystals.

## Refinement

Crystal data, data collection and structure refinement details are summarized in Table 2 ▸.

## Supplementary Material

Crystal structure: contains datablock(s) global, I. DOI: 10.1107/S2414314624004759/hb4471sup1.cif

Structure factors: contains datablock(s) I. DOI: 10.1107/S2414314624004759/hb4471Isup2.hkl

Supporting information file. DOI: 10.1107/S2414314624004759/hb4471Isup3.cml

CCDC reference: 2356945

Additional supporting information:  crystallographic information; 3D view; checkCIF report

## Figures and Tables

**Table 1:** Hydrogen-bond geometry (Å, °)

*D*—H⋯*A*	*D*—H	H⋯*A*	*D*⋯*A*	*D*—H⋯*A*
C17—H17⋯*Cg*3^i^	0.93	2.77	3.598 (4)	148
C18—H18⋯*Cg*5^i^	0.93	2.85	3.661 (4)	146

Symmetry code: (i) 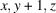 .

**Table 2:** Experimental details

Crystal data	
Chemical formula	C_26_H_18_BrN
*M*_r_	424.32
Crystal system, space group	Monoclinic, *P*2_1_/*c*
Temperature (K)	293
*a*, *b*, *c* (Å)	13.0619 (17), 7.6891 (10), 20.475 (3)
β (°)	106.809 (14)
*V* (Å^3^)	1968.6 (5)
*Z*	4
Radiation type	Mo *K*α
μ (mm^−1^)	2.10
Crystal size (mm)	0.48 × 0.39 × 0.30

Data collection	
Diffractometer	Agilent Xcalibur, Atlas, Gemini
Absorption correction	Analytical (*CrysAlis RED*; Agilent, 2012 ▸)
*T*_min_, *T*_max_	0.432, 0.572
No. of measured, independent and observed [*I* > 2σ(*I*)] reflections	12046, 3999, 3115
*R*_int_	0.037
(sin θ/λ)_max_ (Å^−1^)	0.625

Refinement	
*R*[*F* ^2^ > 2σ(*F* ^2^)], *wR*(*F* ^2^), *S*	0.049, 0.129, 1.05
No. of reflections	3999
No. of parameters	257
H-atom treatment	H atoms treated by a mixture of independent and constrained refinement
Δρ_max_, Δρ_min_ (e Å^−3^)	0.81, −1.40

Computer programs: *CrysAlis PRO* (Agilent, 2012 ▸), *SHELXT* (Sheldrick, 2015*a* ▸), *SHELXL2018/3* (Sheldrick, 2015*b* ▸) and *PLATON* (Spek, 2020 ▸).

## full crystallographic data

### Crystal data

**Table:**

C_26_H_18_BrN	*F*(000) = 864
*M**_r_* = 424.32	*D*_x_ = 1.432 Mg m^−^^3^
Monoclinic, *P*2_1_/*c*	Mo *K*α radiation, λ = 0.71073 Å
*a* = 13.0619 (17) Å	Cell parameters from 3999 reflections
*b* = 7.6891 (10) Å	θ = 3.4–26.4°
*c* = 20.475 (3) Å	µ = 2.10 mm^−^^1^
β = 106.809 (14)°	*T* = 293 K
*V* = 1968.6 (5) Å^3^	Plate, yellow
*Z* = 4	0.48 × 0.39 × 0.30 mm

### Data collection

**Table:**

Agilent Xcalibur, Atlas, Gemini diffractometer	3115 reflections with *I* > 2σ(*I*)
Radiation source: fine-focus sealed tube	*R*_int_ = 0.037
ω scans	θ_max_ = 26.4°, θ_min_ = 3.4°
Absorption correction: analytical (CrysAlis RED; Agilent, 2012)	*h* = −16→14
*T*_min_ = 0.432, *T*_max_ = 0.572	*k* = −9→8
12046 measured reflections	*l* = −25→25
3999 independent reflections	

### Refinement

**Table:**

Refinement on *F*^2^	0 restraints
Least-squares matrix: full	Hydrogen site location: inferred from neighbouring sites
*R*[*F*^2^ > 2σ(*F*^2^)] = 0.049	H atoms treated by a mixture of independent and constrained refinement
*wR*(*F*^2^) = 0.129	*w* = 1/[σ^2^(*F*_o_^2^) + (0.0558*P*)^2^ + 1.6534*P*] where *P* = (*F*_o_^2^ + 2*F*_c_^2^)/3
*S* = 1.05	(Δ/σ)_max_ < 0.001
3999 reflections	Δρ_max_ = 0.81 e Å^−^^3^
257 parameters	Δρ_min_ = −1.40 e Å^−^^3^

### Special details

**Table:**

Geometry. All esds (except the esd in the dihedral angle between two l.s. planes) are estimated using the full covariance matrix. The cell esds are taken into account individually in the estimation of esds in distances, angles and torsion angles; correlations between esds in cell parameters are only used when they are defined by crystal symmetry. An approximate (isotropic) treatment of cell esds is used for estimating esds involving l.s. planes.
Refinement. The N-bound hydrogen atom was located in a difference map and its position was freely refined. The remaining hydrogen atoms were positioned geometrically [C—H = 0.93 Å] and were refined using a riding model, with *U*_iso_(H) = 1.2 or 1.5*U*_eq_(C).

### Fractional atomic coordinates and isotropic or equivalent isotropic displacement parameters (Å^2^)

**Table:**

	*x*	*y*	*z*	*U*_iso_*/*U*_eq_	
Br1	−0.02528 (3)	−0.04566 (6)	0.34351 (2)	0.0715 (2)	
N1	0.4598 (2)	0.3135 (4)	0.41519 (18)	0.0577 (9)	
C3	0.1079 (2)	0.2407 (4)	0.40118 (14)	0.0324 (6)	
C12	0.1886 (2)	0.3680 (4)	0.40287 (14)	0.0315 (6)	
C11	0.2396 (2)	0.3735 (4)	0.35115 (14)	0.0309 (6)	
C5	0.1305 (2)	0.1255 (4)	0.29426 (14)	0.0321 (6)	
C10	0.2127 (2)	0.2523 (4)	0.29757 (14)	0.0306 (6)	
C4	0.0816 (2)	0.1236 (4)	0.34678 (15)	0.0346 (6)	
C15	0.3228 (2)	0.5092 (4)	0.35332 (15)	0.0338 (6)	
C9	0.2637 (2)	0.2517 (4)	0.24464 (15)	0.0396 (7)	
H9	0.317747	0.331703	0.246104	0.048*	
C20	0.4302 (2)	0.4749 (4)	0.38369 (16)	0.0374 (7)	
C2	0.0601 (3)	0.2382 (4)	0.45579 (15)	0.0424 (7)	
H2	0.007271	0.156905	0.455513	0.051*	
C8	0.2351 (3)	0.1373 (4)	0.19247 (16)	0.0467 (8)	
H8	0.269377	0.139879	0.158491	0.056*	
C21	0.5558 (2)	0.2265 (4)	0.41906 (17)	0.0410 (7)	
C13	0.2159 (3)	0.4878 (4)	0.45853 (16)	0.0426 (7)	
H13	0.267045	0.573083	0.460148	0.051*	
C6	0.1034 (3)	0.0083 (4)	0.23773 (16)	0.0426 (7)	
H6	0.049834	−0.073800	0.234413	0.051*	
C7	0.1544 (3)	0.0148 (4)	0.18900 (17)	0.0487 (8)	
H7	0.135678	−0.063095	0.152715	0.058*	
C16	0.2937 (3)	0.6726 (4)	0.32505 (18)	0.0478 (8)	
H16	0.221626	0.697953	0.305740	0.057*	
C26	0.6048 (3)	0.1311 (4)	0.47645 (18)	0.0482 (8)	
H26	0.576252	0.129337	0.513136	0.058*	
C18	0.4752 (3)	0.7621 (4)	0.35375 (18)	0.0501 (8)	
H18	0.526590	0.845360	0.353300	0.060*	
C1	0.0911 (3)	0.3530 (5)	0.50787 (16)	0.0484 (8)	
H1	0.060381	0.347710	0.543532	0.058*	
C19	0.5059 (3)	0.6027 (4)	0.38339 (19)	0.0492 (8)	
H19	0.578117	0.579804	0.403488	0.059*	
C22	0.6002 (3)	0.2280 (5)	0.36512 (19)	0.0512 (8)	
H22	0.567577	0.291351	0.325915	0.061*	
C17	0.3693 (3)	0.7984 (4)	0.32496 (19)	0.0545 (9)	
H17	0.348321	0.906707	0.305481	0.065*	
C14	0.1681 (3)	0.4789 (5)	0.50894 (17)	0.0497 (8)	
H14	0.187045	0.558049	0.544786	0.060*	
C23	0.6920 (3)	0.1364 (5)	0.3695 (2)	0.0620 (10)	
H23	0.721646	0.139989	0.333358	0.074*	
C25	0.6966 (3)	0.0379 (5)	0.4793 (3)	0.0678 (12)	
H25	0.729331	−0.027472	0.517943	0.081*	
C24	0.7402 (3)	0.0409 (5)	0.4254 (3)	0.0717 (12)	
H24	0.801947	−0.021919	0.427468	0.086*	
H55	0.425 (3)	0.265 (6)	0.435 (2)	0.073 (14)*	

### Atomic displacement parameters (Å^2^)

**Table:**

	*U*^11^	*U*^22^	*U*^33^	*U*^12^	*U*^13^	*U*^23^
Br1	0.0704 (3)	0.0804 (3)	0.0719 (3)	−0.0470 (2)	0.0336 (2)	−0.0247 (2)
N1	0.0311 (15)	0.0545 (18)	0.092 (2)	0.0093 (13)	0.0244 (16)	0.0365 (17)
C3	0.0271 (14)	0.0351 (15)	0.0330 (15)	0.0021 (11)	0.0057 (11)	0.0035 (12)
C12	0.0279 (14)	0.0333 (14)	0.0306 (15)	0.0019 (11)	0.0041 (11)	0.0025 (11)
C11	0.0212 (13)	0.0327 (14)	0.0357 (15)	0.0024 (11)	0.0036 (11)	0.0057 (12)
C5	0.0284 (14)	0.0309 (14)	0.0329 (15)	0.0031 (11)	0.0024 (12)	0.0014 (11)
C10	0.0255 (13)	0.0326 (14)	0.0314 (14)	0.0047 (11)	0.0047 (11)	0.0053 (11)
C4	0.0260 (14)	0.0365 (15)	0.0378 (16)	−0.0035 (12)	0.0039 (12)	0.0021 (12)
C15	0.0315 (15)	0.0330 (14)	0.0371 (16)	−0.0015 (12)	0.0103 (13)	0.0024 (12)
C9	0.0374 (16)	0.0413 (17)	0.0415 (17)	0.0049 (13)	0.0136 (14)	0.0070 (13)
C20	0.0315 (15)	0.0360 (15)	0.0465 (18)	0.0015 (12)	0.0140 (13)	0.0089 (13)
C2	0.0389 (17)	0.0494 (18)	0.0401 (17)	−0.0023 (14)	0.0133 (14)	0.0030 (14)
C8	0.052 (2)	0.054 (2)	0.0379 (17)	0.0078 (16)	0.0188 (15)	0.0020 (15)
C21	0.0286 (15)	0.0322 (15)	0.059 (2)	−0.0012 (12)	0.0069 (14)	0.0050 (14)
C13	0.0433 (18)	0.0419 (17)	0.0410 (18)	−0.0080 (14)	0.0098 (14)	−0.0061 (13)
C6	0.0469 (18)	0.0389 (16)	0.0379 (17)	−0.0039 (14)	0.0059 (14)	−0.0037 (13)
C7	0.062 (2)	0.0439 (18)	0.0373 (18)	0.0047 (16)	0.0093 (16)	−0.0058 (14)
C16	0.0389 (17)	0.0378 (17)	0.061 (2)	0.0031 (14)	0.0050 (15)	0.0133 (15)
C26	0.0388 (17)	0.0457 (18)	0.056 (2)	0.0000 (14)	0.0074 (15)	0.0072 (16)
C18	0.051 (2)	0.0384 (18)	0.065 (2)	−0.0136 (15)	0.0232 (18)	0.0003 (15)
C1	0.0504 (19)	0.060 (2)	0.0377 (18)	0.0041 (16)	0.0181 (15)	−0.0013 (15)
C19	0.0313 (16)	0.0508 (19)	0.066 (2)	−0.0044 (14)	0.0145 (16)	0.0070 (17)
C22	0.049 (2)	0.0476 (19)	0.056 (2)	−0.0016 (16)	0.0134 (17)	0.0038 (16)
C17	0.060 (2)	0.0309 (16)	0.070 (2)	−0.0027 (15)	0.0140 (19)	0.0133 (16)
C14	0.057 (2)	0.054 (2)	0.0386 (18)	−0.0039 (16)	0.0151 (16)	−0.0098 (15)
C23	0.065 (2)	0.045 (2)	0.090 (3)	−0.0036 (18)	0.043 (2)	−0.009 (2)
C25	0.045 (2)	0.052 (2)	0.095 (3)	0.0124 (17)	0.002 (2)	0.024 (2)
C24	0.048 (2)	0.052 (2)	0.121 (4)	0.0122 (18)	0.035 (3)	0.006 (2)

### Geometric parameters (Å, º)

**Table:**

Br1—C4	1.896 (3)	C21—C22	1.388 (5)
N1—C20	1.401 (4)	C13—C14	1.353 (5)
N1—C21	1.404 (4)	C13—H13	0.9300
N1—H55	0.78 (4)	C6—C7	1.352 (5)
C3—C4	1.396 (4)	C6—H6	0.9300
C3—C2	1.430 (4)	C7—H7	0.9300
C3—C12	1.432 (4)	C16—C17	1.383 (5)
C12—C11	1.405 (4)	C16—H16	0.9300
C12—C13	1.429 (4)	C26—C25	1.385 (5)
C11—C10	1.405 (4)	C26—H26	0.9300
C11—C15	1.497 (4)	C18—C17	1.366 (5)
C5—C4	1.400 (4)	C18—C19	1.375 (5)
C5—C6	1.428 (4)	C18—H18	0.9300
C5—C10	1.437 (4)	C1—C14	1.392 (5)
C10—C9	1.427 (4)	C1—H1	0.9300
C15—C20	1.386 (4)	C19—H19	0.9300
C15—C16	1.390 (4)	C22—C23	1.371 (5)
C9—C8	1.351 (5)	C22—H22	0.9300
C9—H9	0.9300	C17—H17	0.9300
C20—C19	1.395 (4)	C14—H14	0.9300
C2—C1	1.353 (4)	C23—C24	1.352 (6)
C2—H2	0.9300	C23—H23	0.9300
C8—C7	1.400 (5)	C25—C24	1.381 (6)
C8—H8	0.9300	C25—H25	0.9300
C21—C26	1.376 (5)	C24—H24	0.9300
			
C20—N1—C21	124.8 (3)	C12—C13—H13	119.6
C20—N1—H55	122 (3)	C7—C6—C5	121.0 (3)
C21—N1—H55	113 (3)	C7—C6—H6	119.5
C4—C3—C2	123.6 (3)	C5—C6—H6	119.5
C4—C3—C12	118.0 (2)	C6—C7—C8	120.8 (3)
C2—C3—C12	118.4 (3)	C6—C7—H7	119.6
C11—C12—C13	121.2 (3)	C8—C7—H7	119.6
C11—C12—C3	120.6 (3)	C17—C16—C15	121.5 (3)
C13—C12—C3	118.2 (3)	C17—C16—H16	119.2
C10—C11—C12	120.1 (2)	C15—C16—H16	119.2
C10—C11—C15	120.1 (2)	C21—C26—C25	119.7 (4)
C12—C11—C15	119.8 (2)	C21—C26—H26	120.2
C4—C5—C6	123.7 (3)	C25—C26—H26	120.2
C4—C5—C10	118.1 (2)	C17—C18—C19	120.2 (3)
C6—C5—C10	118.2 (3)	C17—C18—H18	119.9
C11—C10—C9	121.9 (3)	C19—C18—H18	119.9
C11—C10—C5	120.2 (2)	C2—C1—C14	121.1 (3)
C9—C10—C5	118.0 (3)	C2—C1—H1	119.5
C3—C4—C5	123.0 (3)	C14—C1—H1	119.5
C3—C4—Br1	118.7 (2)	C18—C19—C20	120.8 (3)
C5—C4—Br1	118.3 (2)	C18—C19—H19	119.6
C20—C15—C16	118.6 (3)	C20—C19—H19	119.6
C20—C15—C11	120.8 (3)	C23—C22—C21	120.1 (3)
C16—C15—C11	120.6 (3)	C23—C22—H22	119.9
C8—C9—C10	121.3 (3)	C21—C22—H22	119.9
C8—C9—H9	119.4	C18—C17—C16	119.4 (3)
C10—C9—H9	119.4	C18—C17—H17	120.3
C15—C20—C19	119.4 (3)	C16—C17—H17	120.3
C15—C20—N1	118.9 (3)	C13—C14—C1	120.9 (3)
C19—C20—N1	121.7 (3)	C13—C14—H14	119.5
C1—C2—C3	120.6 (3)	C1—C14—H14	119.5
C1—C2—H2	119.7	C24—C23—C22	121.3 (4)
C3—C2—H2	119.7	C24—C23—H23	119.3
C9—C8—C7	120.7 (3)	C22—C23—H23	119.3
C9—C8—H8	119.6	C24—C25—C26	120.7 (4)
C7—C8—H8	119.6	C24—C25—H25	119.7
C26—C21—C22	119.1 (3)	C26—C25—H25	119.7
C26—C21—N1	119.3 (3)	C23—C24—C25	119.1 (4)
C22—C21—N1	121.5 (3)	C23—C24—H24	120.4
C14—C13—C12	120.8 (3)	C25—C24—H24	120.4
C14—C13—H13	119.6		
			
C4—C3—C12—C11	−0.2 (4)	C11—C15—C20—N1	−2.8 (5)
C2—C3—C12—C11	178.7 (3)	C21—N1—C20—C15	148.5 (3)
C4—C3—C12—C13	−179.9 (3)	C21—N1—C20—C19	−32.9 (5)
C2—C3—C12—C13	−1.0 (4)	C4—C3—C2—C1	178.5 (3)
C13—C12—C11—C10	178.9 (3)	C12—C3—C2—C1	−0.3 (4)
C3—C12—C11—C10	−0.8 (4)	C10—C9—C8—C7	−0.3 (5)
C13—C12—C11—C15	−1.1 (4)	C20—N1—C21—C26	144.7 (4)
C3—C12—C11—C15	179.2 (2)	C20—N1—C21—C22	−38.2 (5)
C12—C11—C10—C9	−179.0 (3)	C11—C12—C13—C14	−178.4 (3)
C15—C11—C10—C9	1.0 (4)	C3—C12—C13—C14	1.3 (5)
C12—C11—C10—C5	1.9 (4)	C4—C5—C6—C7	−179.1 (3)
C15—C11—C10—C5	−178.1 (2)	C10—C5—C6—C7	0.8 (4)
C4—C5—C10—C11	−2.0 (4)	C5—C6—C7—C8	−0.2 (5)
C6—C5—C10—C11	178.1 (3)	C9—C8—C7—C6	0.0 (5)
C4—C5—C10—C9	178.8 (2)	C20—C15—C16—C17	1.9 (5)
C6—C5—C10—C9	−1.0 (4)	C11—C15—C16—C17	−178.4 (3)
C2—C3—C4—C5	−178.8 (3)	C22—C21—C26—C25	−0.4 (5)
C12—C3—C4—C5	0.0 (4)	N1—C21—C26—C25	176.8 (3)
C2—C3—C4—Br1	1.3 (4)	C3—C2—C1—C14	1.6 (5)
C12—C3—C4—Br1	−179.9 (2)	C17—C18—C19—C20	1.0 (5)
C6—C5—C4—C3	−179.1 (3)	C15—C20—C19—C18	0.4 (5)
C10—C5—C4—C3	1.1 (4)	N1—C20—C19—C18	−178.3 (3)
C6—C5—C4—Br1	0.8 (4)	C26—C21—C22—C23	−0.5 (5)
C10—C5—C4—Br1	−179.03 (19)	N1—C21—C22—C23	−177.5 (3)
C10—C11—C15—C20	−86.9 (3)	C19—C18—C17—C16	−0.9 (6)
C12—C11—C15—C20	93.1 (3)	C15—C16—C17—C18	−0.5 (6)
C10—C11—C15—C16	93.3 (4)	C12—C13—C14—C1	−0.1 (5)
C12—C11—C15—C16	−86.7 (4)	C2—C1—C14—C13	−1.3 (5)
C11—C10—C9—C8	−178.3 (3)	C21—C22—C23—C24	1.1 (6)
C5—C10—C9—C8	0.8 (4)	C21—C26—C25—C24	0.6 (6)
C16—C15—C20—C19	−1.8 (5)	C22—C23—C24—C25	−0.9 (6)
C11—C15—C20—C19	178.5 (3)	C26—C25—C24—C23	0.0 (6)
C16—C15—C20—N1	176.9 (3)		

### Hydrogen-bond geometry (Å, º)

**Table:**

*D*—H···*A*	*D*—H	H···*A*	*D*···*A*	*D*—H···*A*
C17—H17···*Cg*3^i^	0.93	2.77	3.598 (4)	148
C18—H18···*Cg*5^i^	0.93	2.85	3.661 (4)	146

Symmetry code: (i) *x*, *y*+1, *z*.
